# Serum Free Fatty Acids and G-Coupled Protein Receptors Are Associated With the Prognosis of Epithelial Ovarian Cancer

**DOI:** 10.3389/fonc.2022.777367

**Published:** 2022-06-17

**Authors:** Lili Zhang, Xiangzhong Zhao, Huijun Chu, Han Zhao, Xiaoying Lai, Jing Li, Teng Lv

**Affiliations:** ^1^ Department of Nutrition, The Affiliated Hospital of Qingdao University, Qingdao, China; ^2^ Medical Research Center, The Affiliated Hospital of Qingdao University, Qingdao, China; ^3^ Department of Gynaecology, The Affiliated Hospital of Qingdao University, Qingdao, China; ^4^ Department of Pathology, The Affiliated Hospital of Qingdao University, Qingdao, China; ^5^ Department of Nephrology, The Affiliated Hospital of Qingdao University, Qingdao, China

**Keywords:** free fatty acids, ovarian cancer, FFAR (free fatty acid receptor), G-protein coupled receptor, prognosis

## Abstract

**Purpose:**

Fatty acid metabolism plays key role in cancer development, and free fatty acid receptors (FFARs) are involved in many cancers. However, the correlation between serum free fatty acids (FFAs)/FFARs levels and ovarian cancer (OC) prognosis remains largely unclear.

**Methods:**

A retrospective review of 534 primary OC patients and 1049 women with benign ovarian tumors was performed. Serum FFA levels data were extracted from the electronic medical record system. Repeated FFA results of 101 OC patients treated with standard chemotherapy were collected. The effects of FFAs on cells migration were evaluated in OC cell lines by Transwell assay. Gene Expression Profiling Interactive Analysis (GEPIA) was used to compare FFAR mRNA expression levels in cancer and noncancer tissues. Kaplan-Meier (KM) plotter was employed to analyze their prognostic values. SPSS 23.0 and Graphpad prism 7.0 software was used for analysis and graph construction.

**Results:**

FFA levels in the serum of epithelial ovarian cancer (EOC) women were higher than in women with benign ovarian tumors independent of pathology, tumor stage,and grade. FFA levels decreased gradually after chemotherapy. FFAs enhanced the migration of OVCAR3 cells. FFAR1 mRNA expression was lower in OC cells than in control cells. FFAR3 was related to a better prognosis, and FFAR4 was related to poor prognosis in TP-53wild-type and mutated type OC, while FFAR1 and FFAR2 were related to a better prognosis in TP53 wild-type OC but FFAR2 was related to a poor prognosis in TP53-mutant OC.

**Conclusion:**

The FFA levels are increased in OC and decreased with chemotherapy. High expression of FFARs was related to the prognosis of OC. The prognostic value of different FFARs differs depending on whether it is a TP53 wild or TP53 mutant ovarian cancer.Targeting FFARs may be an attractive treatment strategy for EOC.

## Introduction

Ovarian cancer (OC) is the third common malignant tumor of the female reproductive system, has the eighth highest incidence among female cancer,and accounts for 4.4% of cancer-related deaths in females (1). Epithelial cancers account for 90% of all cases and encompass a heterogeneous group of malignancies comprised of five main histotypes: high-grade serous ovarian cancer (HGSOC), endometrioid ovarian cancer (ENOC), clear cell ovarian cancer (CCOC), mucinous ovarian cancer (MOC), and low-grade serous (LGSOC) (2, 3), the most common of which are serous carcinomas (4). Epithelial cancers are typically more aggressive than nonepithelial malignancies since most serous carcinomas are diagnosed at an advanced stage, which accounts for the low survival rates (4). Improving prevention and early detection strategies has improved the outcome, but the prognosis is still not ideal, and the 5-year survival rate is generally approximately 30% for advanced-stage OC (5). This high mortality rate of OC can be attributed to extensive peritoneal metastasis at the time of diagnosis (6, 7). The omentum is mainly composed of adipocytes, which have been considered to promote the initial homing of tumor cells and provide fatty acids to cancer cells, fueling rapid tumor growth (8). Free fatty acids (FFAs) are intermediate products of lipid mobilization that result principally from lipolysis and the breakdown of triglycerides (TGs) (9). In addition to the function of energy sources, FFAs also have the key functions of receptor signaling, gene expression and system energy homeostasis regulation under various physiological conditions (10–12). Serum FFAs include short-chain fatty acids (SCFAs), medium-chain fatty acids (MCFAs), and long-chain fatty acids (LCFAs); in cancer patients, most of FFAs are acetic acid (32%), hexadecanoic acid (40%) and octadecanoic acid (9%). Among them, acetic acid level is lower and hexadecanoic acid/octadecanoic acid is higher in cancer patients than in healthy controls (13).The free fatty acid receptor (FFAR) family is comprised of FFAR1–4, formerly known as GPR40, GPR43, GPR41, and GPR120, respectively; FFAR1 and FFAR4 are both receptors for LCFAs, and FFAR2 and FFAR3 are both receptors for SCFA (14–16). FFARs are expressed in several cancer cells, and their effect depends on the cell type and tumor type. Data on the role of FFARs in OC are limited. Ovarian cell lines and animal models showed that ovarian cells express mRNA for FFAR1 and FFAR4, and FFAR agonist/antagonist affect cell growth (17, 18). Clinical studies of FFAs and prognosis are limited. A previous study showed that serum FFA levels were elevated even when TGs and total cholesterol (TCHO) were normal in tumor patients, especially OC patients (19). In clinical work, we found that in OC patients, the serum FFA level was dramatically decreased after chemotherapy. Here, we wanted to determine the serum FFA levels in OC patients and the role of FFARs in the prognosis of OC.

## Materials and Methods

### Patients

This study was approved by the Medical Ethics Committee of Affiliated Hospital of Qingdao University (Shandong, China). Data for patients diagnosed with primary epithelial ovarian cancer (EOC) From October 2013 to June 2020 were extracted from the electronic medical record system of The Affiliated Hospital of Qingdao University. Patients with recurrent OC or other non-ovarian cancer were excluded from the patient group. The control group was composed of women who underwent surgery for a benign ovarian mass, who had never had cancer. For both groups, patients with diabetes mellitus, hyperlipidemia, abnormal thyroid function, stroke, cardiovascular disease and pregnancy were excluded. Clinical data [sex, age, body mass index (BMI), histopathological and International Federation of Gynecology and Obstetrics (FIGO) stage (20)] were obtained. The general data of the two groups are shown in **Table 1**.

**Table 1:** Comparison of patients with OC and benign ovarian tumors.

Characteristic	OC patients (n = 516)	Cyst patients (n = 1049)	u (P value)	OR	Multivariate analysis	
					95% CI	P value
Average age (years)	54 (48,63)	48 (37,53)	<0.001	1.09	1.08-1.10	<0.001
BMI (kg/m^2^)	23.04 (20.81,25.22)	23.92 (21.67,26.48)	0.000	0.89	0.86-0.92	0.000
FFA (mmol/L)	0.51 (0.38,0.67)	0.44 (0.32,0.60)	0.000	2.91	1.89-4.48	0.000

### FFA Testing

The enzyme endpoint method was applied to measure the serum FFA level. The FFAs that we measured were nonesterified fatty acids. A Beckman Coulter AU5800 biochemical analyzer (Beckman Coulter, Inc. CA, USA) was used for analysis.

### Cell Lines and Reagents

The human OC cell lines OVCAR3 were purchased from Procell Life Science & Technology Co., Ltd. (Wuhan, China). Cells were cultured in Dulbecco’s modified Eagle’s medium (Gibco, NY, USA) containing 10% fetal bovine serum (FBS, Gibco) and 1% penicillin/streptomycin. The cells were maintained in an incubator at 37°C with 5% CO_2_. Sodium oleate and sodium palmitate were obtained from Kunchuang Biotechnology (Xi’an, China).

### Transwell Assay

The migration capability of cells was detected with a 24-well Transwell system (polycarbonate filter inserts with pore size of 8 μm and density of 1X10^5^ pores/cm^2^) (Corning Costar, MA, USA). Briefly, 1 × 10^5^ cells in 200 µl serum-free DMEM were seeded into the upper chamber. Sodium oleate (OA), sodium palmitate (PA) or FBS was added to the lower chamber as a chemoattractant at final concentrations of 200 µM, 100 µM, and 10%, respectively, in 500 μL serum-free medium. After 48 h of incubation, the cells were fixed with 4% paraformaldehyde for 10 min and stained with 0.1% crystal violet for 30 min. Nonmigrating cells were removed by wiping the upper chamber with a cotton swab. Migrated cells in the lower chamber were photographed and quantified in five random fields under a microscope at ×100 magnification (Nikon).

### FFAR Gene Expression Analysis

Gene Expression Profiling Interactive Analysis (GEPIA, http://gepia.cancer-pku.cn/) is a web-based tool that contains The Cancer Genome Atlas (TCGA) and Genotype-Tissue Expression (GTEx) data (21). In this study, we analyzed the relative expression of FFAR genes in OC and normal samples. The log2FC cutoff was 1, and the p-value cutoff was 0.01.

### FFAR Gene Expression and Survival Analysis

Kaplan-Meier Plotter (https://kmplot.com/analysis/) can be used to perform real-time multivariate survival analysis according to genes in available transcriptomic cohorts (22). In this study, it was used to analyze associations between FFARs and overall survival (OS), progression-free survival (PFS) of OC patients (516 patients with TP53 mutations and 102 patients with wild-type TP53); the OC patients were divided into high and low expression groups based on the median values for mRNA expression and assessed by K-M survival curves with hazard ratios (HRs) and 95% confidence intervals (CIs) and log-rank p-values. A p-value <0.05 was considered to indicate a statistically significant difference.

### Statistical Analysis

The data and figures were mainly processed with SPSS 23.0 and GraphPad Prism 7.0. The mean and 25th and 75th percentile values [M (P25, P75)] were used to express the results when the measurement data did not conform to a normal distribution. Count data, which did not conform to a normal distribution, were described according to the frequency and percentage. The Mann-Whitney U test was used for comparisons of two groups, the Kruskal-Wallis h test was used for comparisons among three or more groups, and the chi-square test was used for intergroup comparisons of count data. One-way ANOVA was adopted for statistical analysis. The Kaplan-Meier method was used to estimate OS and PFS. Differences in survival outcomes were assessed by the log-rank test. Results were presented as HRs and 95% CIs.Statistical significance was accepted at the P <0.05 level.

## Results

### Serum FFA Levels Were Elevated in OC Cases

FFA levels were recorded before surgery and chemotherapy. In our study, serum FFAs were collected from 1049 benign ovarian tumor patients and 516 OC patients. The main baseline characteristics of the patients are summarized in **Table 1**. The cancer patients were older than the cyst patients (54 vs 48 years, P < 0.001). The BMI in the cancer group was 23.04 kg/m2, which was lower than that in the cyst group (23.92 kg/m2, P < 0.001). Compared with the cyst group, the cancer group had a higher FFA level (0.51 vs 0.44 mmol/L, P < 0.001). As shown in **Table 2**,We performed further correlation analysis and after adjusting for age and BMI, each unit of FFA values generated a 191% risk of OC [OR (95% CI): 2.91 (1.89-4.48), P < 0.001].

**Table 2:** Survival analyses of FFAR1 and FFAR4 in ovarian cancer (Kaplan–Meier plotter).

	HR (95%CI)	P	Median OS (months)	
			Low expression	High expression
FFAR1-wild-type				
PFS	0.36 (0.12-1.14)	0.071	9.72	19.09
OS	0.3 (0.09-0.96)	0.033	19.02	41.89
FFAR1-TP53 mutation				
PFS	1.16 (0.77-1.77)	0.48	14.19	16.07
OS	1.48 (0.97-2.25)	0.068	37.03	30.17
FFAR2-wild-type				
PFS	0.54 (0.32-0.91)	0.019	12.67	19.43
OS	0.59 (0.33-1.04)	0.063	32.5	48.27
FFAR2-TP53 mutation				
PFS	1.32 (1.03-1.69)	0.026	21.43	16.93
OS	1.52 (1.17-1.97)	0.0014	50.69	37.32
FFAR3-wild-type				
PFS	0.7 (0.41-1.19)	0.18	13.86	19.43
OS	0.47 (0.26-0.84)	0.0097	34.87	52.07
FFAR3-TP53 mutation				
PFS	0.75 (0.57-0.97)	0.027	17.6	18.3
OS	0.8 (0.61-1.05)	0.1	39.63	48.2
FFAR4-wild-type				
PFS	2.92 (1.06-8.05)	0.031	19.09	9.72
OS	3.73 (1.11-12.57)	0.024	65.17	23.37
FFAR4-TP53 mutation				
PFS	1.83 (1.23-2.71)	0.0024	17.41	7.1
OS	1.41 (0.96-2.06)	0.076	37.93	29.08

### FFA Levels Are Not Associated With Pathological Types, Stage or Grade

**Figure 1:**
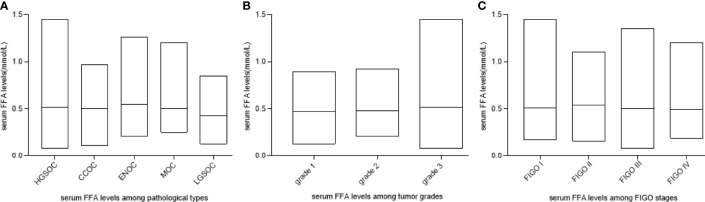
FFA levels among OC patients with different pathological types, stages and grades. **(A)** FFA levels among pathological types (P > 0.05). **(B)** FFA levels among tumor grades (P > 0.05) **(C)** FFA levels among FIGO stages (P > 0.05). HGSOC, high-grade serous ovarian cancer (HGSOC); ENOC, endometrioid ovarian cancer; CCOC, clear cell ovarian cancer; MOC, mucinous ovarian cancer; LGSOC, low-grade serous.

We assessed whether the FFA level was different among tumors with different pathological types, stages and grades. As shown in **Figures 1A–C**, there was no significant difference among patients with different pathological types, tumor grades and FIGO classifications (P>0.05).

### Serum FFA Level Decreases After Chemotherapy

**Figure 2:**
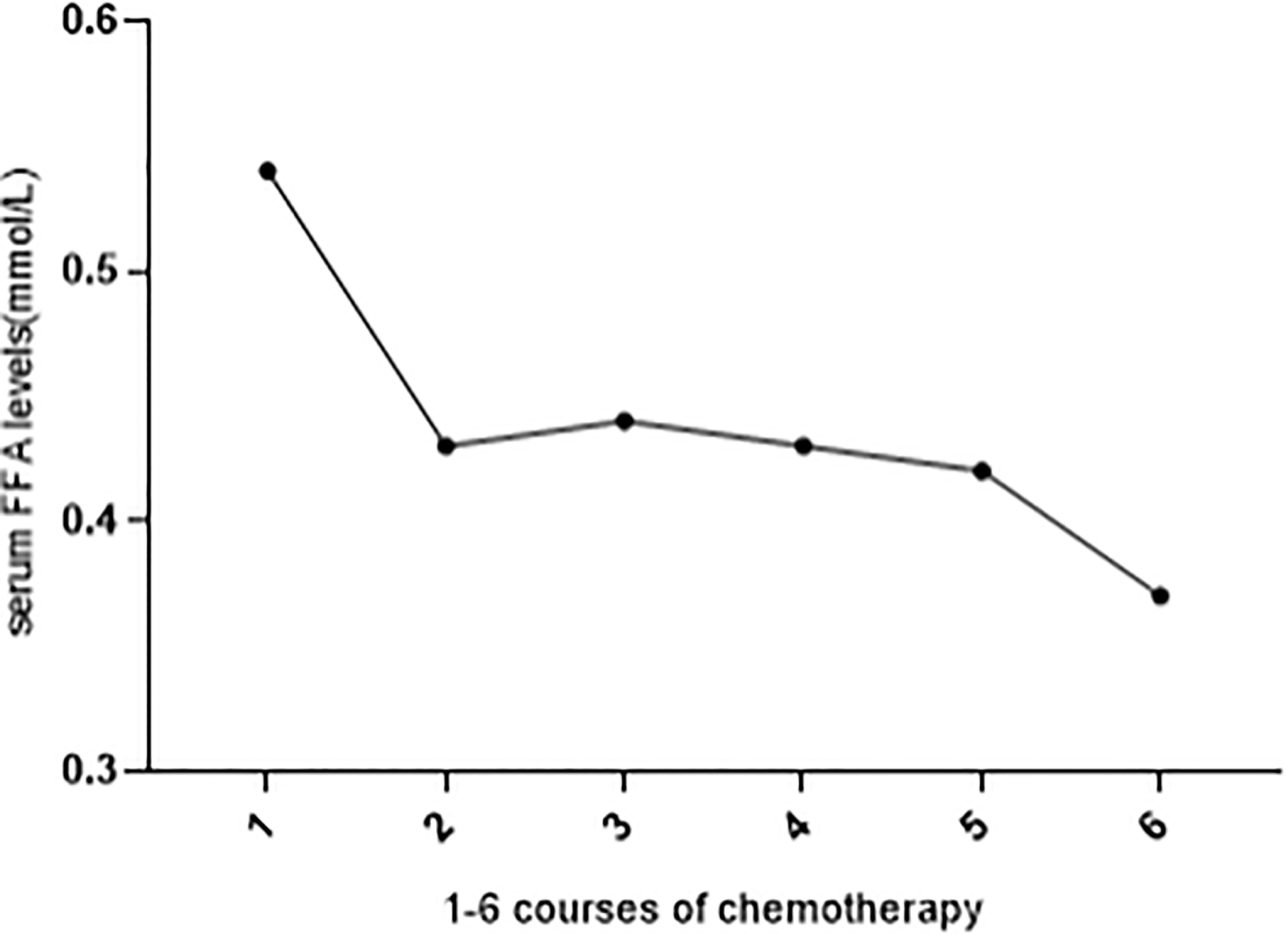
FFA levels before 1-6 courses of chemotherapy. Serum FFA level was significantly decreased during 1-6 courses of chemotherapy (compared with 1 course, all other courses P < 0.05).

We observed that after chemotherapy, there was a fluctuation in the FFA level. Among 516 OC patients, 106 patients received standard chemotherapy in our hospital and had FFA data before chemotherapy. **Figure 2** shows the trend of FFA changes before 1-6 courses of chemotherapy. The level of FFAs decreased gradually during chemotherapy, and the difference between 1 course and 2-6 courses was statistically significant (P<0.05).

### FFAs Promote OVCAR3 Cell Migration

**Figure 3:**
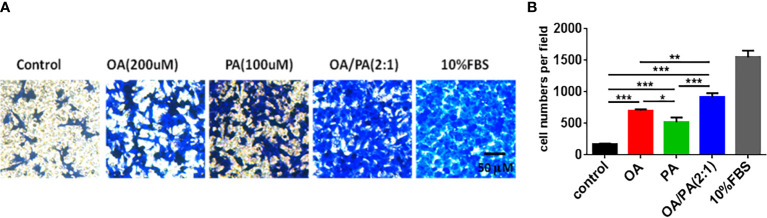
Effects of OA and PA stimulation on ovarian carcinoma cells migration. **(A)** OVCAR3 cells were treated with OA and PA as described in methods, migrated cells were stained with crystal violet and photographed. Shown are representative images (100× magnification). **(B)** The quantitative data of cells migration ability. The data are presented as means ± SEM, n = 5, *P < 0.05, **P < 0.01, ***P < 0.001.

To explore the effect of FFAs on OC cell tumorigenesis, a Transwell assay was adopted to analyze the response of OVCAR3 cells to PA(100umol/L), OA(200umol/L) and OA:PA (2:1) stimulation. **Figure 3** showed that the migration of OVCAR3 cells was obviously increased in response to PA, OA and OA:PA. Moreover, OA and PA displayed synergistic effects on OVCAR3 cell migration, indicating the important role of FFAs in tumor migration *in vitro*.

### The mRNA Expression Level of FFAR1 Was Lower in the OC Group

**Figure 4:**
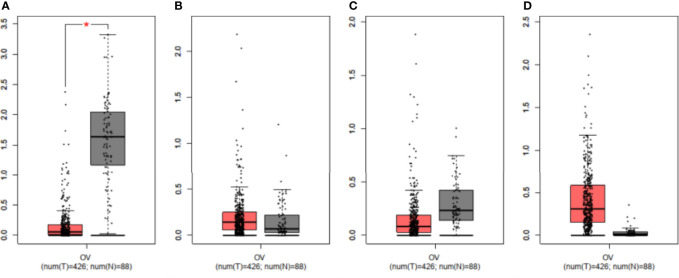
Validation of mRNA expression levels of **(A)** FFAR1, **(B)** FFAR2, **(C)** FFAR3, **(D)** FFAR4 in OC tissues and normal ovary tissues using GEPIA. The box plots were based on 426 OC (showed in red)and 88 normal ovary samples(showed in grey). *P < 0.01 was considered statistically significant.

We compared the mRNA levels of FFARs using online GEPIA datasets (426 OC and 88 normal ovary samples). **Figure 4** showed that the FFAR1 level was lower in OC tissues than in normal ovary tissues (P<0.01), while there were no statistical significance in the FFAR2, FFAR3, and FFAR4 levels between OC and normal control samples.

### FFAR Is Highly Associated With the Prognosis of OC

**Table 2** shows that high expression of FFAR1 was related to better OS (41.89 vs 19.02 months) in wild-type OC. High expression of FFAR2 was related to better PFS (19.43 vs 12.67 months) in patients with wild-type OC but was related to poor PFS (16.93 VS 21.43 months), poor OS (37.32 vs 50.69 s) in those with TP53 mutation. High expression of FFAR3 was related to better OS (52.07 vs 34.87) in wild-type patients and better PFS (18.3 vs 17.6) in TP53-mutated patients. High expression of FFAR4 was related to poor PFS (9.72 vs 19.09 months), OS (23.37 vs 65.17 months) in wild-type OC and poor PFS (7.1 vs 17.41 months) in TP53-mutated OC. In conclusion,FFAR1-3 is related to a better prognosis, and FFAR4 is related to a poor prognosis in wild-type OC; FFAR2,4 are related to a poor prognosis and FFAR3 is related to a better prognosis in those with TP53-mutated OC.

## Discussion

A previous review showed that overweight (BMI of 25–29.9 kg/m2) and obesity (BMI ≥ 30 kg/m2) appears to increase the risk of OC (23), while our study found that when BMI is within the normal range (BMI of 18.5-24.9 kg/m2), OC has lower BMI than benign ovarian tumor. We found that the FFA level elevated in patients with OC and gradually decreased after chemotherapy, FFAs promote OVCAR3 cell migration, indicating that FFAs play a role during cancer development and progression, which was clearly demonstrated in several studies in related fields. Recent advances in proteomics and metabolomics have deepened our understanding of the role of fatty acid metabolism in determining the fate of cancer cells (24). The TG/FFA cycle participates in various metabolic and physiological processes and provides signaling molecules in cells. Fatty acid metabolism not only supports energy production but also provides necessary substrates for membrane synthesis and metabolic processes through β-oxidation, which is crucial to neogenesis (25, 26).

We found that the FFA level was not related to the pathological type, grade or FIGO stage of OC, while after chemotherapy, the FFA level declined dramatically, which can be explained by the decrease in the number of tumor cells and the diminished requirement of lipid metabolism after chemotherapy since abundant FAs in cancer cells support the rapid growth and proliferation of cancer cells (27).

The roles of FFARs in human cancers and cancer cells are different. In some tumors, FFAR1 and FFAR4 have similar effects, and the activation of FFAR1 and FFAR4 can promote the proliferation and migration of breast cancer cells and enhance the growth of colon cancer cells (28–30). In some tumors, FFAR1 and FFAR4 have the opposite effect; for example, FFAR1 activation inhibits the motile activity of pancreatic cancer and osteosarcoma cells, while FFAR4 activation promotes these activities (31, 32). FFAR1 activation promotes lung cancer and melanoma and prostate cancer, while FFAR4 activation inhibits these processes (33–36). FFAR1 activation inhibits the migration and invasion in fibrosarcoma cells (37), whereas FFAR4 activation stimulates the migration and invasion of esophageal cancer cells (38). FFAR4 activation can promote radiation resistance and chemotherapy resistance (39, 40). FFAR2 and FFAR3 act as tumor suppressors in colon and breast cancer; the loss of FFAR2 promotes colon tumorigenesis (41–43). FFAR2 is expressed in a human cervical cancer cell line and plays a protective role in cervical cancer, leukemia, and oral squamous cell carcinoma (44–46)

Previous studies on the effect of FFARs on OC cells are controversial. One article reported that an FFAR agonist resulted in growth inhibition and was associated with an alteration in energy metabolism, while another article found that an FFAR1 antagonist inhibited the proliferation of OC cells (17, 18). Munkarah et al. found that high-grade serous carcinoma specimens have significantly high expression of FFAR1 than normal ovary specimens and that FFAR1 expression was related to stage; higher expression was noted in advanced-stage disease (18). From the GEPIA data, we found that the mRNA levels of FFAR1/3 were lower in tumors, while those of FFAR2/4 were higher in tumors, but only FFAR1 showed a significant difference. KM plotter analysis showed that in wild-type OC, FFAR1-3 expression is related to a better prognosis, and FFAR4 is related to a poor prognosis; in TP53-mutant OC, FFAR2,4 are related to a poor prognosis, whereas FFAR3 is related to a better prognosis. FFAR2 has opposite effects, while FFAR3 and FFAR4 have similar effects on the prognosis of P53 wild and mutant OC. To our knowledge, FFARs are significant, independent prognostic factors in OC.

Our research has certain limitations. First, the number of cell lines and animal models was limited. Second, we did not assess the protein expression of FFARs in OC tissues, and further study needs to be done.

In summary, we found that serum FFAs were drastically downregulated after chemotherapy and that FFARs may be attractive potential therapeutic targets for OC. Further studies are required to study the mechanism and elucidate the link between the signaling and metabolic cross-talk occurring through FFARs and OC.

## Conclusions

In summary, high FFA levels in the serum may be an indicator of OC, and FFARs are related to the prognosis of OC. This study may therefore provide new insight to OC monitoring and prognosis evaluation.

## Data Availability Statement

The datasets presented in this study can be found in online repositories. The names of the repository/repositories and accession number(s) can be found in the article/supplementary material.

## Ethics Statement

The studies involving human participants were reviewed and approved by The Medical Ethics Committee of The Affiliated Hospital of Qingdao University (Shandong, China). Written informed consent for participation was not required for this study in accordance with the national legislation and the institutional requirements. Written informed consent was not obtained from the individual(s) for the publication of any potentially identifiable images or data included in this article.

## Author Contributions

HZ, XL, and JL collected and organized the data, XZ and HC performed the statistical analysis and generated the figures. LZ wrote the paper. TL designed the study and wrote the paper. All authors contributed to the article and approved the submitted version.

## Conflict of Interest

The authors declare that the research was conducted in the absence of any commercial or financial relationships that could be construed as a potential conflict of interest.

## Publisher’s Note

All claims expressed in this article are solely those of the authors and do not necessarily represent those of their affiliated organizations, or those of the publisher, the editors and the reviewers. Any product that may be evaluated in this article, or claim that may be made by its manufacturer, is not guaranteed or endorsed by the publisher.

## Abbreviations

HGSOC, high-grade serous ovarian cancer; ENOC, endometrioid ovarian cancer; CCOC, clear cell ovarian cancer; MOC, mucinous ovarian cancer; LGSOC, low-grade serous; FFAR,free fatty acid receptor; FFA, free fatty acid; OC, ovarian cancer; GEPIA, Gene Expression Profiling Interactive Analysis; KM, Kaplan-Meier; EOC, epithelial ovarian cancer; TGs, triglycerides; SCFAs, short-chain fatty acids; MCFAs, medium-chain fatty acids; LCFAs, long-chain fatty acids; TCHO, total cholesterol; EOC, epithelial ovarian cancer; OA, Sodium oleate; PA, sodium palmitate; GEPIA, Gene Expression Profiling Interactive Analysis; TCGA, Cancer Genome Atlas; GTEx, Genotype-Tissue Expression; OS, overall survival; PFS, progression-free survival; HRs, hazard ratios; CIs, confidence intervals; FIGO, International Federation of Gynecology and Obstetrics.
